# Neuropatía craneal múltiple secundaria a meningitis tuberculosa y tuberculoma intracraneal en una adolescente

**DOI:** 10.7705/biomedica.8106

**Published:** 2026-08-20

**Authors:** Daniela Pabón, Christian Andrés Rojas, Carolina Barco

**Affiliations:** 1 Departamento de Pediatría, Hospital Universitario del Valle, Cali, Colombia Departamento de Pediatría Hospital Universitario del Valle Cali Colombia; 2 Departamento de Pediatría, Universidad del Valle, Cali, Colombia Universidad del Valle Departamento de Pediatría Universidad del Valle Cali Colombia

**Keywords:** tuberculosis meníngea, enfermedades de los nervios craneales, tuberculoma, Mycobacterium tuberculosis, antituberculosos, Tuberculosis, meningeal, cranial nerve diseases, tuberculoma, Mycobacterium tuberculosis, antitubercular agents

## Abstract

La neurotuberculosis -una forma grave de tuberculosis extrapulmonar- representa, aproximadamente, el 1 % de los casos y es causa frecuente de discapacidad neurológica en los países en desarrollo. La meningitis tuberculosa, la forma más común, afecta principalmente a niños menores de 5 años, individuos inmunodeprimidos y pacientes con HIV, aunque también puede presentarse en adolescentes sanos. La detección temprana y el tratamiento oportuno son esenciales para reducir la morbilidad y la mortalidad.

La paciente, una adolescente de 17 años, presentó cefalea holocraneana, fiebre, visión borrosa y somnolencia, con progresión a parálisis de los pares craneales III y VI, sin los signos clásicos de la meningitis temprana. En la resonancia magnética (RM) cerebral se observaron imágenes nodulares hiperintensas con T2-FLAIR de la cabeza del núcleo caudado izquierdo e intraventricular en el *septum pellucidum* con realce en anillo después de la administración del medio de contraste, sugestivas de tuberculomas, mientras que en el líquido cefalorraquídeo se encontró pleocitosis linfocítica, hiperproteinorraquia e hipoglucorraquia. La confirmación diagnóstica se logró mediante la prueba Xpert™ MTB/ RIF en líquido cefalorraquídeo, la cual fue positiva para *Mycobacterium tuberculosis.* La evolución clínica mejoró con el tratamiento antituberculoso y corticosteroides.

Con este caso se destaca la importancia de considerar la neurotuberculosis en aquellos pacientes con síntomas neurológicos subagudos, incluso en adolescentes sin factores evidentes de riesgo. La identificación temprana y el tratamiento adecuado son fundamentales para prevenir las secuelas neurológicas graves y mejorar el pronóstico.

## Abstract (en)

Neurotuberculosis -a severe form of extrapulmonary tuberculosis- accounts for approximately 1% of all cases and is a frequent cause of neurological disability in developing countries. Tuberculous meningitis, the most common form, primarily affects children under 5 years of age, immunocompromised individuals, and HIV-positive patients, although it can also occur in otherwise healthy adolescents. Early detection and timely treatment are essential to reduce morbidity and mortality.

The patient, a 17-year-old adolescent girl, presented with holocranial headache, fever, blurred vision, and somnolence, progressing to paralysis of the III and VI cranial nerves, without classical signs of early meningitis. Brain magnetic resonance imaging revealed hyperintense nodular lesions on T2-FLAIR sequences in the head of the left caudate nucleus and intraventricularly in the *septum pellucidum,* with ring enhancement after contrast administration, consistent with tuberculomas. Cerebrospinal fluid analysis showed lymphocytic pleocytosis, elevated protein levels, and low glucose concentration. Diagnostic confirmation was achieved through a positive Xpert™ MTB/RIF test on cerebrospinal fluid for *Mycobacterium tuberculosis.* Clinical improvement was observed following antituberculous therapy and corticosteroids.

This case highlights the importance of considering neurotuberculosis in patients with subacute neurological symptoms, even in adolescents without evident risk factors. Early identification and appropriate treatment are crucial to prevent severe neurological sequelae and improve outcomes.

La tuberculosis es una enfermedad endémica en la mayoría de los países en desarrollo. La neurotuberculosis es la infección de las meninges, el cerebro o la médula espinal ^1^; representa el 1 % de los casos de tuberculosis activa y es la forma más letal de la enfermedad; además, es una causa frecuente de discapacidad neurológica en los países de escasos recursos ^2^. La meningitis tuberculosa es la forma más prevalente, representa del 70 al 80 % de los casos de neurotuberculosis y es una de las formas más graves de la tuberculosis extrapulmonar ^3^.

La neurotuberculosis puede afectar a cualquier grupo etario, aunque los niños menores de 5 años, las personas coinfectadas con HIV y los individuos inmunodeprimidos constituyen la población de mayor riesgo ^4^.

Su manifestación clásica es una enfermedad meningítica subaguda, aunque el signo típico de meningitis -rigidez de la nuca- generalmente está ausente en las etapas tempranas. En esta fase, los síntomas son inespecíficos e incluyen poca ganancia de peso, fiebre, apatía, cefalea y vómitos. Cuando se desarrollan los signos neurológicos característicos de la tuberculosis meníngea avanzada -irritación meníngea, coma, convulsiones, signos de presión intracraneal elevada, parálisis de los nervios craneales, hemiparesia y trastornos del movimiento- el pronóstico del paciente se torna más reservado ^1^^,^^5^.

El diagnóstico y el tratamiento tempranos son importantes, ya que el retraso en el diagnóstico se asocia con un peor pronóstico como secuelas neurológicas, trastornos neurocognitivos y muerte ^2^. Se recomienda la administración de medicamentos antituberculosos por 9 a 12 meses y corticoesteroides sistémicos por 4 semanas como terapia complementaria ^6^.

Se reporta el caso de una paciente de 17 años, previamente sana, con tuberculosis del sistema nervioso central, con una neuropatía craneal múltiple. Este informe de caso se elaboró siguiendo las pautas CARE (CAse REport) ^7^.

## Caso clínico

Se trata de una paciente de raza negra de 17 años, estudiante de bachillerato, que ingresó al servicio de urgencias de un hospital de Cali (Colombia) por una cefalea holocraneana de quince días de evolución, de intensidad moderada, que mejoraba parcialmente con la administración oral de analgésicos (acetaminofén, 4 g al día) y se asociaba con despertares nocturnos. También refirió malestar general, mialgias, fiebre no cuantificada, visión borrosa y somnolencia en la última semana. Negó haber presentado síntomas respiratorios o haber tenido contacto con pacientes sintomáticos de enfermedades respiratorias. No se identificaron antecedentes relevantes personales ni familiares.

Anteriormente había consultado a urgencias en atención primaria, en donde se consideró que los síntomas correspondían a una migraña y se le dio manejo ambulatorio. En la tercera semana de la persistencia de los síntomas, y ante la evidencia de midriasis del ojo derecho -identificado por la cuidadora-, consultó nuevamente al servicio de urgencias en donde se descartó dengue y se le practicó una tomografía de cráneo sin contraste en la que se observó una imagen nodular intraaxial hiperdensa de 14 x 10 mm en el núcleo caudado izquierdo, que había provocado el colapso del asta anterior del ventrículo lateral del mismo lado.

En el examen neurológico de ingreso, la paciente estaba alerta y orientada. Presentaba parálisis completa del III par craneal derecho y del VI par craneal izquierdo, con fuerza muscular conservada. En el resto del examen neurológico no se observaron alteraciones. Fue valorada por oftalmología y no se evidenció papiledema.

Entre los estudios practicados, se encontró lo siguiente:

- linfopenia: 940 células por mm^3^, sin otras alteraciones;
- prueba cutánea de tuberculina para la detección de infección tuberculosa PPD *(Purified Protein Derivative),* negativa;
- anticuerpos contra HIV 1 y 2 con prueba rápida inmunocromatográfica, negativos;
- prueba serológica RPR *(Rapid Plasma Reagin)* de tamizaje para sífilis, negativa;
- *Toxoplasma gondii:* IgG, positiva, e IgM, negativa;
- niveles de inmunoglobulinas séricas, normales, y
- disminución del número de linfocitos T (LTCD3+, 493 por mm^3^; LTCD4+, 273 por mm^3^; LTCD8+, 209 por mm^3^).

Se practicó resonancia magnética (RM) cerebral con contraste, en la que se identificaron dos imágenes nodulares hiperintensas con T_2_-FLAIR *(Fluid Attenuated Inversion Recovery)* en la cabeza del núcleo caudado izquierdo e intraventricular en el *septum pellucidum* con realce en anillo después de la administración del medio de contraste; además, se evidenció realce paquimeníngeo en la cisterna perimesencefálica y en el tentorio del cerebelo (figura 1).

**Figure 1:**
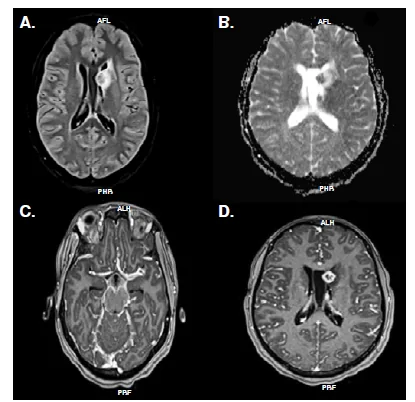
(A) Corte axial con T_2_-FLAIR en el que se observa imagen hipointensa con hiperintensidad periférica del núcleo caudado izquierdo de 14 x 12 x 16 mm. (B) Corte axial en secuencia de *apparent diffusion coefficient* (ADC) con hipointensidad gangliobasal izquierda, (C y D) Corte axial T1 con contraste, en el que se observa realce leptomeníngeo en las cisternas de la base.

En el segundo día de hospitalización, se practicó punción lumbar con los siguientes resultados: examen citoquímico: hiperproteinorraquia (325 mg/dl), hipoglucorraquia (26,9 mg/dl), pleocitosis con predominio de linfocitos (91 por mm^3^), mononucleares (1 por mm^3^; polimorfonucleares (8 por mm^3^); lactato deshidrogenasa, elevada (82,2 UI/L). Asimismo, se realizó una prueba con el panel Biofire Filmarray™ para meningitis y encefalitis, con resultado negativo, y el cultivo de líquido cefalorraquídeo fue negativo para bacterias aerobias (cuadro 1).

**Cuadro 1:** Exámenes de laboratorio practicados durante la hospitalización

Día 1
HIV, anticuerpos 1 y 2, negativos (prueba rápida inmunocromatográfica) Prueba no treponémica, no reactiva IgA, 111,3 mg/dl; IgG, 857, 3 mg/dl; IgM, 56,80 mg/dl; IgE, 72,77 kUI/L (kilounidades internacionales) *Toxoplasma gondii*: IgG, 80,80 UI/ml; IgM, 0.11 COI (*cut-off index*); PPD, 0 mm
Día 2
Citoquímico de líquido cefalorraquídeo: Proteínas: 325 mg/dl Glucosa: 26,9 mg/dl Linfocitos: 91 por mm_3_; mononucleares: 1 por mm^3^; polimorfonucleares: 8 por mm^3^ LDH, 82,2 UI/L, elevada Tinta china para hongos: negativa; cultivo para bacterias aerobias de líquido cefalorraquídeo: negativo; cultivo para hongos: negativo Panel Biofire Filmarray™ para meningitis o encefalitis por virus, bacterias y levaduras: no se detectaron. *Crytococcus neoformans*, antígeno, no se detectó. Cultivo para micobacterias: positivo a las 5 semanas de incubación
Día 5
• GenXpert™ en líquido cefalorraquídeo para *Mycobacterium tuberculosis,* positivo
Día 15
HTLV I y II, 0,02, no reactivo Linfocitos totales: 858 por mm3 LT CD3, 493 por mm3, bajo (valores de referencia: 1088,1-2087,9) LT CD3, 57.4 %, normal (valores de referencia: 53.3-75.3) LT CD3+CD4+, 273 por mm3, bajo (valores de referencia: 639.5 1278.5) LT CD3+CD4+, 31.8 %, normal (valores de referencia: 30.7-46) LT CD3+CD8+, 209 por mm3, bajo (valores de referencia: 332-774.2) LT CD3+CD8+, 24,3%, normal (valores de referencia: 16.2-28.7) LTCD4+/TCD8+, 1,3
Día 30
Citoquímico de líquido cefalorraquídeo: Proteínas: 127 mg/dl Glucosa: 26,9 mg/dl Eritrocitos: 0 por mm^3^; linfocitos: 94 por mm^3^; mononucleares: 1 por mm3; polimorfonucleares: 5 por mm^3^ LDH en líquido cefalorraquídeo: 15,3 UI/L *Crytococcus neoformans*, no se detectó antígeno. GenXpert™ en líquido cefalorraquídeo para *Mycobacterium tuberculosis*, no se detectó.

Con base en los datos clínicos, imagenológicos y de laboratorio, en el tercer día se decidió iniciar tratamiento tetraconjugado y esteroides intravenosos (cuadro 2) ante la sospecha de tuberculosis del sistema nervioso central.

**Cuadro 2:** Medicamentos administrados durante la hospitalización

Día 2 hasta el día 14 y reinicio el día 23
Rifampicina (150 mg) - isoniacida (75 mg): 4 tabletas cada 24 horas Pirazinamida (400 mg): 4 tabletas cada 24 horas Etambutol (400 mg): 3 tabletas cada 24 horas
Día 15 hasta el día 22
• Rifampicina (150 mg) más isoniacida (75 mg) más pirazinamida (400 mg) más etambutol (275 mg): 4 tabletas cada 24 horas.
Día 2 y por 4 semanas más
• Protocolo para dexametasona: 0,4 g/kg al día, luego disminución paulatina hasta suspender su administración

A las 48 horas de iniciado el tratamiento, se obtuvieron resultados positivos para *Mycobacterium tuberculosis* en la prueba Xpert™ MTB/RIF Ultra en líquido cefalorraquídeo.

En la RM de control practicada una semana después de iniciado el tratamiento, se observó persistencia del tuberculoma -aunque de menor tamaño- y reducción del realce leptomeníngeo. Se evidenció un infarto cerebral frontal derecho y otro en el ganglio basal izquierdo. También, se realizó video electroencefalográfico de 24 horas en el que se encontraron signos de encefalopatía leve sin actividad epileptiforme ictal o interictal.

Dado que no fue posible determinar la sensibilidad *M. tuberculosis* debido a la baja carga bacilar, y ante el riesgo de secuelas neurológicas graves, se practicó una nueva punción lumbar. En esta se encontró mejoría de los parámetros del examen citoquímico y se informó un resultado negativo de la prueba con Xpert™ MTB/RIF Ultra.

Considerando la evolución clínica favorable y la mejoría de los estudios de laboratorio e imagenológicos, se decidió continuar el tratamiento de primera línea con isoniacida, rifampicina, pirazinamida y etambutol (cuadro 2).

Una semana después de iniciado el tratamiento, se resolvió la somnolencia, aunque persistía la alteración de la memoria de trabajo y la parálisis del III par (figura 1).

### Aspectos éticos

El caso cuenta con el consentimiento informado de la paciente, de acuerdo con los formatos establecidos por la *British Medical Journal* (BMJ *Case Reports)* y en cumplimiento de la Resolución 1830 del Ministerio de Salud y Protección Social.

## Discusión

La meningitis tuberculosa es una de las formas más graves de la tuberculosis extrapulmonar y una causa importante de morbimortalidad en las regiones endémicas, como Colombia.

Entre el 2012 y el 2013, se reportaron 1146 casos de tuberculosis meníngea en Colombia; la población más afectada estuvo en el rango etario de los 25 a los 34 años y se presentó principalmente en hombres ^8^. En pediatría, la mayoría de los casos se presenta en menores de 5 años, según los estudios de India y Sudáfrica ^2^^,^^9^.

El presente caso desafía la tendencia epidemiológica al tratarse de una adolescente, lo cual subraya la necesidad de considerar el diagnóstico de meningitis tuberculosa en las poblaciones sin factores de riesgo, como infección por HIV, desnutrición o inmunodeficiencia, y con cuadros neurológicos subagudos.

La presentación clínica en pediatría puede ser variada e inespecífica y depende de la ubicación, y el tamaño y número de las lesiones ^10^. Según el estudio de Ramzan *et al.*^11^, los síntomas más comunes incluyen cefalea (86 %) y vómito (69 %), además de la afectación del nervio óptico y de los nervios craneales III y VI en el 28 % de los casos.

Por su parte, Mane *et al.*^2^ reportaron que el 77 % de los pacientes presentaron crisis epilépticas. No obstante, en nuestro caso, la paciente no presentó crisis epilépticas clínicas ni electroencefalográficas.

En la serie de casos de Jamoussi *et al.*^1^, los pacientes manifestaron regresión en el neurodesarrollo, emesis, irritabilidad, convulsiones, cefalea y accidentes cerebrovasculares. En la paciente del presente caso, la cefalea subaguda progresó a alteración del estado de conciencia y neuropatías craneales (pares III y VI). Estos hallazgos son coherentes con la literatura publicada, en la que las neuropatías craneales se reportan del 20 al 52 % de los casos ^12^, generalmente, con compromiso de un solo par craneal, siendo más frecuente el compromiso del nervio *abducens* (VI par) ^13^.

En el estudio de Li *et al.,* el 22 % de los pacientes presentó compromiso de dos pares craneales, comúnmente en combinaciones como el nervio oculomotor y el nervio óptico, el nervio óptico y el nervio auditivo, o el nervio óptico y el nervio *abducens*^12^. Esto difiere de la paciente del presente estudio, en el cual se identificó compromiso de los pares craneales III y VI.

En relación con el pronóstico y la evolución, el estudio de An Wen *et al.*^14^ reportó que el 85,7 % de los pacientes con parálisis de pares craneales lograron una recuperación completa. Sin embargo, también señalaron que la parálisis de estos nervios constituye un factor predictor de secuelas neurológicas.

En la etapa temprana, los signos meníngeos no son frecuentes. Sin embargo, en el estudio de Ramzan *et al.*^11^, se reportó que el 51 % presentó rigidez de nuca, y en el de Farinha *et al.*^15^ se informó meningismo en el 47 % de los pacientes. En nuestro caso, la paciente no presentó signos meníngeos en ninguna etapa de la enfermedad.

El diagnóstico de meningitis tuberculosa puede ser desafiante debido a la poca sensibilidad y al tiempo de la obtención de los resultados de los métodos convencionales. En el estudio de van Well *et al.,* solo en el 12 % de los casos se aisló M. *tuberculosis* en el cultivo de líquido cefalorraquídeo ^9^.

La detección de ADN de *M. tuberculosis* en el líquido cefalorraquídeo mediante la PCR múltiple mejora la sensibilidad y la especificidad de los métodos tradicionales. En la literatura médica, se reportan sensibilidades que oscilan entre el 70 y el 90 % para la PCR múltiple o la PCR-Luminex, con especificidades cercanas al 99-100 % cuando se compara con los métodos convencionales, como la tinción de Ziehl-Neelsen o el cultivo de líquido cefalorraquídeo ^16^^,^^17^. En nuestro caso, la prueba con GenXpert™ permitió detectar trazas del agente patógeno, con lo que fue posible confirmar el diagnóstico.

El estudio de Mane *et al.*^2^^)^ sugirió que la proteinorraquia del líquido cefalorraquídeo (> 100 mg/dl) y la resistencia a los medicamentos se asociaban con un desenlace fatal, mientras que el realce meníngeo, los exudados basales y un recuento de linfocitos mayor del 90 % en el líquido cefalorraquídeo lo hacían con secuelas funcionales graves.

En este caso, la paciente presentó un recuento de linfocitos en el líquido cefalorraquídeo de 91 por mm^3^, proteínas por encima de 100 mg/dl y realce meníngeo; sin embargo, después del egreso, tuvo una evolución clínica favorable y recuperación de la autonomía en las actividades de la vida diaria.

En cuanto a la PPD *(Purified Protein Derivative),* un resultado negativo en un paciente con tuberculosis puede explicarse por la poca sensibilidad de la prueba, inmunosupresión, errores técnicos o variabilidad biológica. Este resultado destaca la necesidad de combinar los métodos diagnósticos para mejorar la precisión y no descartar la tuberculosis tras un resultado negativo ^18^. También, es posible obtener un resultado negativo en las etapas tempranas de la enfermedad, dado que la respuesta inmunológica medida por la PPD puede no haberse desarrollado completamente. En nuestro caso, se descartó infección por HIV y se solicitaron estudios adicionales para inmunodeficiencias primarias.

Los tuberculomas cerebrales pueden aparecer en cualquier parte del cerebro. Clásicamente, se describen supratentoriales en los adultos e infratentoriales en pediatría; pueden ser solitarios, pero frecuentemente son múltiples y pueden aparecer aislados o asociados a meningitis tuberculosa ^19^.

En la RM, la apariencia del tuberculoma cerebral depende de la etapa de evolución y de la presencia o ausencia de necrosis caseosa; la apariencia típica solo se encuentra en el 34 % de los casos ^19^. En el presente caso se reportaron características típicas de tuberculoma en dos imágenes nodulares, una en el núcleo caudado izquierdo y otra intraventricular.

La meningitis tuberculosa puede complicarse con arteritis y generar infartos. Esta enfermedad afecta principalmente la parte anterior del tálamo, el núcleo caudado y la cápsula interna (zona tuberculosa) ^20^^,^^21^. En el estudio de Kalita *et al.,* el 50 % de los pacientes presentaba infarto en la zona tuberculosa ^22^. En el caso de la paciente del presente reporte, en la RM cerebral de control se observaron dos infartos en los ganglios basales, asociados con un déficit motor focal transitorio. Sin embargo, se observó una notable mejoría tras el inicio de la terapia antituberculosa y esteroidea. Según Thee *et al.,* el 42 % de los pacientes en estadio II tiene recuperación completa. Estos autores también mencionan que las secuelas más frecuentes son la espasticidad, el retraso cognitivo y la epilepsia ^23^.

En cuanto al tratamiento, la Organización Mundial de la Salud (OMS) recomienda una fase intensiva de dos meses con isoniacida, rifampicina, pirazinamida y estreptomicina, seguida por una fase de continuación con isoniacida y rifampicina ^24^. No obstante, el etambutol ha sido incluido en el esquema primario en varios contextos debido a su efecto esterilizante cuando se combina con otros fármacos antituberculosos de primera línea, así como por su mayor disponibilidad en comparación con la estreptomicina. Esta elección está alineada con las directrices nacionales para el manejo de la tuberculosis ^25^.

## Conclusiones

Este caso subraya la importancia de considerar el diagnóstico de meningitis tuberculosa en aquellos pacientes procedentes de áreas endémicas con síntomas neurológicos subagudos o crónicos, incluyendo la presencia de una neuropatía craneal múltiple, incluso en poblaciones no típicamente afectadas.

El diagnóstico oportuno, apoyado en pruebas moleculares como el GenXpert™ y en las neuroimágenes, junto con un manejo adecuado, puede mejorar significativamente los desenlaces clínicos.
